# Distinguishing bipolar disorder from unipolar depression in general practice: A systematic review

**DOI:** 10.1080/13814788.2026.2733396

**Published:** 2026-09-18

**Authors:** Docteur Scheherazade Niktabe, Catherine Massoubre

**Affiliations:** a General Practitioner, Lyon, France; b Department of Psychiatric Emergency, Université Jean Monnet, Saint Étienne, France

**Keywords:** Bipolar disorder, major depressive disorder, risk factors, diagnostic errors, general practice

## Abstract

**Objective:**

Bipolar disorder affects approximately 1% of the population, although some studies report prevalence rates of up to 6%. Distinguishing it from unipolar depression remains challenging, with diagnostic delays often exceeding ten years. This systematic review aims to identify clinical and historical features of depressive episodes that support the earlier detection of bipolar disorder in general practice.

**Methods:**

A systematic review was conducted in accordance with PRISMA guidelines. The PubMed database was searched using MeSH terms. Study quality was assessed using the Newcastle-Ottawa Scale and the PRISMA checklist.

**Results:**

Of the 525 articles identified, 36 met the inclusion criteria. Early age at onset, recurrent episodes, family history of bipolar disorder, cyclothymic temperament, suicide attempts, postpartum depression, psychotic features and mixed episodes emerged as the most consistently associated indicators of bipolar disorder. Other clinical characteristics showed more heterogeneous or limited associations. Although individual features lack sufficient predictive value when considered in isolation, their combined presence may enhance clinical suspicion and facilitate earlier identification of bipolar disorder in primary care settings.

**Conclusion:**

This review identified a combination of clinical and historical features of depressive episodes that help distinguish between bipolar disorder and unipolar depression in general practice. Identifying these features through a structured clinical interview, as a complement to existing screening tools such as the Mood Disorder Questionnaire, may help reduce diagnostic delays and improve outcomes.

## KEY MESSAGES

- Early onset, recurrence, family history, cyclothymic temperament, suicide attempts, postpartum depression, psychotic features, and mixed episodes differentiate bipolar from unipolar depression.
- These specific clinical features highly enhance suspicion when clustering together.
- A structured clinical interview in general practice may help reduce diagnostic delays and improve outcomes.

## Introduction

A major depressive episode is defined in the Diagnostic and Statistical Manual of Mental Disorders, Fifth Edition (DSM-5), as a period of markedly depressed mood lasting at least 14 days and representing a clear change from previous functioning [1]. Such episodes may occur in the context of unipolar depression or as part of bipolar disorder, where they alternate with manic or hypomanic episodes. This review includes studies published before 2013, prior to the DSM-5. At that time, mixed states were defined only when full criteria for both manic and depressive episodes were met, and bipolar disorder was largely diagnosed on the basis of overt mania, limiting the identification of patients initially presenting with depression. Differentiating unipolar from bipolar depression remains a major diagnostic challenge in primary care. Bipolar disorder affects approximately 1% of the population, although some studies report prevalence rates of up to 6% [2], and is often diagnosed late due to the complexity of establishing an accurate diagnosis in routine clinical practice. This diagnostic delay negatively impacts patients’ quality of life, contributing to financial instability, unemployment, social withdrawal, and is associated with an increased risk of suicide [3] and greater public healthcare costs [4]. While unipolar depression is typically managed with antidepressants, bipolar disorder requires long-term treatment with mood stabilisers. The inappropriate use of antidepressants in undiagnosed bipolar disorder may precipitate manic switches or induce rapid cycling, both of which are linked to poorer clinical outcomes [5,6]. Given the limited access to outpatient psychiatric care, general practitioners play a key role in the early detection of bipolar disorder. This systematic review aims to identify clinical and historical features that may help differentiate bipolar from unipolar depression in general practice.

## Methods

This systematic review followed the PRISMA 2020 guidelines  [7]. A PubMed search targeted observational studies, systematic reviews, and meta-analyses in English or French. To enhance completeness, research reports from funded studies were also included. The literature search included all studies available in PubMed up to July 2025.

The search strategy was as follows: (Depressive Disorder/diagnosis [MeSH] OR depressive disorder/psychology [MeSH] OR ‘unipolar depression*’ [Title/Abstract] OR ‘depressive disorder*’ [Title/Abstract] OR ‘major depressive disorder*’ [Title/Abstract] OR ‘depressive mood disorder*’ [Title/Abstract]) AND (Bipolar Disorder/diagnosis [MeSH] OR bipolar disorder/psychology [MeSH] OR ‘bipolar disorder*’ [Title/Abstract] OR ‘Manic Depressive Psychosis’ [Title/Abstract] OR ‘bipolar depression*’ [Title/Abstract] OR ‘bipolar mood disorder*’ [Title/Abstract] OR ‘bipolar*’ [Title/Abstract]) AND (diagnostic error [MeSH] OR ‘diagnostic error*’ [Title/Abstract] OR misdiagnosis [Title/Abstract] OR differential diagnosis [MeSH] OR ‘differential diagnosis’ [Title/Abstract] OR ‘differential diagnoses’ [Title/Abstract] OR predictor* [Title/Abstract] OR ‘clinical features’ [Title/Abstract])

We included only studies comparing bipolar and unipolar depression based on clinical or historical criteria. Studies limited to neurobiological markers or past manic/hypomanic episode identification were excluded. Articles available only as abstracts, or lacking statistical analysis, were also excluded after full-text review. The PRISMA flow diagram is shown in supplementary Figure 1.

The methodological quality of each included study was assessed using the Newcastle-Ottawa Scale for observational studies [8], its adapted version for cross-sectional designs [9], and the PRISMA checklist for systematic reviews and meta-analyses [7]. Study selection, data extraction, and quality assessment were conducted by a single investigator.

Primary criteria were selected based on their clinical relevance as supported by existing scientific literature. A qualitative synthesis grouped studies by criterion, including only those with relevant statistical results. Eligible results were those demonstrating a statistically significant association (*p* < 0.05), typically expressed as an odds ratio (OR) or relative risk (RR).

Multivariate analyses, being more robust, were prioritised. Univariate results were reported only when multivariate data were unavailable. Studies were classified according to the direction of the association with bipolar disorder: supporting bipolar disorder, non-significant, or supporting unipolar depression. Data extraction for all studies was carried out by a single investigator.

This study did not distinguish between bipolar disorder type I and type II, as the aim was to identify bipolar disorder as a whole—regardless of subtype—during a depressive episode. A criterion was considered discriminative if it enabled the differentiation of either bipolar I or II from unipolar depression. Anxiety disorder—including generalised anxiety disorder, panic disorder, phobias, and agoraphobia—was grouped under a single criterion and considered present if any subtype showed a statistically significant association with bipolar disorder. ‘Personality disorder’ was considered relevant if any subtype showed a significant association.

## Results

The search strategy yielded 525 articles. After title and abstract screening, 484 were excluded for not comparing clinical or historical features between unipolar and bipolar depression. Of the 41 full-text articles assessed for eligibility, 36 met the inclusion criteria. Three were excluded due to abstract-only availability, and two lacked statistical analysis.

The 39 primary criteria identified in this review were organised following the structure of Table 2. General information included sociodemographic characteristics such as sex, marital status, education level, and occupational status. Personal history focused on the course of illness, including recurrence of depressive episodes and a history of suicide attempts. Family history referred to psychiatric conditions among relatives, particularly bipolar disorder, depressive episodes, substance use disorders, suicide attempts, and anxiety disorders.

Episode characteristics addressed features of depressive episodes, including age at onset and episode severity. Episode types included specific clinical presentations. Postpartum depression referred to depressive episodes occurring after childbirth. Psychotic depression was defined by the presence of delusions or hallucinations during depressive episodes. Atypical depression included symptoms such as hypersomnia, hyperphagia or weight gain, and mood reactivity. Mixed depression referred to depressive episodes associated with concurrent manic or hypomanic symptoms.

Symptomatology included core depressive symptoms and associated features, such as suicidal ideation, psychomotor retardation or agitation, sleep disturbances, appetite or weight changes, irritability, anxiety, asthenia, concentration difficulties, and anhedonia.

Temperamental traits included cyclothymic temperament, characterised by lifelong mood instability with frequent fluctuations, and hyperthymic temperament, characterised by a persistent pattern of increased energy, activity, and positive mood. Finally, psychiatric comorbidities were reported, including substance use disorders, anxiety disorders, ADHD, personality disorders, eating disorders, and autism spectrum disorders. Response to antidepressant treatment was also reported, including treatment failure and antidepressant-induced manic switch.

Table 1 provides an overview of the study design, sample size, and year of publication, whereas Appendix A presents the quality assessment scores.

**Table 1.** Characteristics of studies selected.

First author, Year, [ref]	Design	Sample size
Scott, 2017, [25]	**Case-control study**	**100**
Päären, 2014, [26]	**Cohort study**	**287**
Benazzi, 2008, [27]	**Cross-sectional study**	**560**
Leonpacher, 2015, [28]	**Cross-sectional study**	**1228**
Akiskal, 1983, [20]	**Cohort study**	**206**
Angst, 2013, [30]	**Cross-sectional study**	**5635**
Wu, 2024, [31]	**Cross-sectional study**	**1463**
Weinstock, 2009, [32]	**Cross-sectional study**	**13753**
Benazzi, 2005, [33]	**Cross-sectional study**	**602**
Hantouche, 1998, [34]	**Cross-sectional study**	**250**
Ratheesh, 2017, [35]	**Meta-analysis**	**56 studies included**
Galvão, 2013, [36]	**Cross-sectional study**	**104**
Perlis, 2006, [37]	**Cross-sectional study**	**1551**
Moreno, 2012, [8]	**Cross-sectional study**	**7124**
Rastelli, 2013, [39]	**Cross-sectional study**	**96**
Serra, 2015, [40]	**Cohort study**	**334**
Benazzi, 2004, [41]	**Cross-sectional study**	**73**
Benazzi, 2006, [42]	**Cross-sectional study**	**650**
Schaffer, 2010, [43]	**Cross-sectional study**	**4612**
Patella, 2019, [44]	**Cross-sectional study**	**241**
Gan, 2011, [45]	**Cohort study**	**268**
Rybakowski, 2005, [46]	**Cross-sectional study**	**880**
Kiejna, 2006, [47]	**Cross-sectional study**	**246**
Xiang, 2013, [48]	**Cross-sectional study**	**1487**
Inoue, 2015, [49]	**Cross-sectional study**	**448**
Parker, 2013, [50]	**Cross-sectional study**	**352**
Kim, 2020, [51]	**Cohort study**	**291 721**
Takeshima, 2008, [52]	**Cohort study**	**139**
Mitchell, 2011, [53]	**Cross-sectional study**	**366**
Mendlowicz, 2005, [54]	**Cross-sectional study**	**153**
Scott, 2013, [55]	**Cross-sectional study**	**308**
Morishita, 2020, [56]	**Cross-sectional study**	**346**
Musliner, 2018, [57]	**Cohort study**	**91 587**
Pfennig, 2016, [58]	**Cohort study**	**3021**
Li, 2012, [23]	**Cohort study**	**3944**
Angst, 2003, [12]	**Cohort study**	**559**

**Table 2.** Distinguishing bipolar from unipolar depression: Results from included studies.

	Criteria	Significant results in favour of bipolar disorder	Significant results in favour of unipolar disorder	Non-significant results
**General information**	Sex	Female: **35**,**36** Male: 16,**25**,33,**42**	Female: 15,**16,**23,25**,28**,**29**	11,**12,**14,18,19,20,**26,**27, 30,34,37,38,40,41,43
	Marital status: married		33	19,23,24,**28**,34,35,41,**45**
	Education level	low: 23,43		24,25,**28**,29,34,**40,**41,**45**
	Professionally active			24,25,**28**,34,35,41,**45**
**Personal history**	Recurrence of depressive episodes	**12**,**13,15**,**16,**18,19,21,**22,**24,27,**28**,29,32,33,**34,36,37,**38,**42**		**26,**30,35
	Suicide attempts	15,**16,17,**19,23,25,**28**,33,**35**,**43**		11,**13,**24,**34,**38
**Family history**	Bipolar disorder	10,**12**,14,**15,**18,20,21,**22,25,29,**31,32,**35**,**42**		**11,34,**37,**40**
	Depressive episodes	23,**42**		10,11,20,21,25,**35**,40
	Addictions	23		10,11,**35**,**40,42**
	Suicide attempts	25		40,35
	Anxiety disorders	**45**		25,40,**42**
**Episode characteristics**	Early age at first episode	**12**,14,**15,**18,19,20,21**,22,**24,**25,26,**27,**30,**31,32,**33,34,**35**,36,45**		**28,29,**37,38,40
	Severity of episodes	**22,29,42**		**16,**19,24,35,40,41
**Episode types**	Postpartum depression	14,31		24,32
	Psychotic depression	**13**,14,**15**,**16,**20,30,31,32,**33,36,42**		10,11,18,24,**26,**27,**37,38**
	Atypical depression	10,15,18,27		24,**37**
	Mixed depression	**12,13**,**15,**18,21,27,**34,37**		**38**
**Symptomatology**	Suicidal ideation	13,**17,**19,23,**28,**33,**43**		11,**16,**24,**27,**29,38
	Psychomotor retardation	**13**,14,**38**		10,11,16,21,23,**27,28,**29,**43**
	Psychomotor agitation	**27,28**		**13**,**16,**21,29,31,38,**43**
	Hyperphagia and/or weight gain	23,**27,**31,32,**33**		13,**28,**30,**34,**38,**43**
	Weight loss and/or anorexia	29	**16**	13,23,**27,28,**38,**43**
	Hypersomnia	13,14,19,23,**27,30,**31,32,**33**		**28,**38,**43**
	Insomnia	23	**16**,19,**22**	13,**27,28,**29,38,**43**
	Concentration difficulties	**27,**38		11,13,**17,**19,23,24,**28,43**
	Asthenia		30	11,13,**17,**19,24,**27,28,38,43**
	Anxiety	16,**28**	15	29,31
	Irritability	23,**28,30**		
	Anhedonia	**43**		11,13,23,24,**28,**38
**Temperaments**	Cyclothymic	10,19,**25,**31,32,**39,41**		**45**
	Hyperthymic	19,**25**,31,32		**39,41**
**Comorbidities**	Alcohol and/or drug addiction	23,25,**28**,30,**35**,**42,45**		11**,13,**15,19,21,24,**29,36,40,43**
	Anxiety disorders	**16,20,**23,**28**	14,**36,40**	11,13,21,24,30,37,**42,43**
	Eating disorder (ED)	15		11,13**,16,**24,**36,42**
	Personality disorders	**16,**23,**35**		15,**36,42**
	ADHD	**11**		15,**36,42,43**
	Autism Spectrum Disorder (ASD)			**36,42**
**Response to antidepressant treatment**	Failure	31,**44**		32
	Manic switch	10**,13,**14,15,19,**34**		

Bold text indicate multivariate analyses.

ED: eating disorders; ADHD: Attention Deficit Hyperactivity Disorder; ASD: Autism Spectrum Disorder Early age at onset, recurrent episodes, family history of bipolar disorder, cyclothymic temperament, suicide attempts, postpartum depression, psychotic features and mixed episodes emerged as the most consistently associated indicators of bipolar disorder.

## Discussion

Several key clinical criteria point towards bipolar disorder rather than unipolar depression: an early age of onset, a family history of bipolar disorder, a high frequency of recurrence, mixed or psychotic features, postpartum depression, a cyclothymic temperament, and a history of suicide attempts. Conversely, factors such as episode severity, atypical depression, a hyperthymic temperament, sociodemographic variables, and psychiatric comorbidities (e.g. ADHD, anxiety, personality disorders, ASD, eating disorders, substance use disorders) do not reliably differentiate bipolar from unipolar depression.

An early age of onset remains one of the most robust markers of bipolar disorder. A large-scale, 11-year national study demonstrated that an early age at the first episode is an independent predictor of subsequent diagnostic conversion [10], while a large international cohort associated childhood onset with greater familial vulnerability [11]. Episode recurrence is also significantly higher in bipolar disorder, with 20-year follow-up data showing a recurrence rate twice that of unipolar depression [12]. Family history serves as another major indicator of bipolar disorder, with heritability estimated at 85% in a landmark twin study [13].

Psychotic features strongly suggest bipolar disorder, carrying a prevalence 4.6 times higher than in unipolar depression [14]. The postpartum period represents another critical window: between 21.4% and 54% of women presenting with postpartum depression actually meet the criteria for bipolar disorder [15], and the risk of relapse is substantially elevated in patients with a pre-existing diagnosis [16]. Furthermore, mixed features are three times more frequent in bipolar disorder [17]. However, the DSM-5 removed irritability, distractibility, and psychomotor agitation from the definition of mixed episodes [1], whereas several authors argue that including these specific criteria enhances the discrimination between the two disorders [18].

Regarding temperamental markers, a study of 1,723 patients identified cyclothymia as the only reliable indicator of bipolarity [19], its characteristic mood fluctuations justifying its inclusion within the subsyndromic bipolar spectrum by some authors [20]. Finally, a history of suicide attempts is nearly four times more frequent in bipolar disorder than in unipolar depression, a phenomenon closely linked to higher levels of impulsivity [21,22].

To date, only three studies have evaluated treatment failure as a marker of bipolar disorder. While a multivariate analysis of over 3,500 patients linked treatment resistance to an increased risk of bipolarity [23] and a meta-analysis confirmed the limited efficacy of antidepressants [24], this criterion lacks specificity in primary care settings. Indeed, in general practice, treatment resistance often stems from poor adherence or subtherapeutic dosing of antidepressants within the context of unipolar depression.

### Strengths and limitations

A major strength of this review is its emphasis on high-level evidence, integrating data from meta-analyses and large cohorts to ground the clinical discussion in robust findings. Additionally, the approach focuses on criteria that are easily accessible in general practice, thereby facilitating their application in routine clinical settings.

However, several methodological limitations must be acknowledged. First, the selection, extraction, and quality assessment of data were performed by a single investigator, which introduces a potential risk of selection bias or subjectivity. This risk was minimised by the strict, a priori application of predefined eligibility criteria.

Second, the lack of consensus regarding clinical thresholds (such as the exact cut-off for age of onset or number of recurrences) limits the standardisation of predictive tools in primary care. Moreover, episode recurrence and past suicide attempts are retrospective markers that inherently reflect a significant diagnostic delay.

Third, the lack of association with episode severity may be due to the heterogeneity of the assessment scales used across studies (e.g. BPRS, HDRS, MADRS, PHQ-9), which limits direct comparability. Finally, although postpartum depression is widely documented in the literature, our findings may suffer from a selection bias due to the underrepresentation of this specific population in the included data.

### Implications for general practice

General practitioners, who stand on the front lines amidst challenging barriers to specialist care, play a pivotal role in early detection. While several screening tools are available for diagnosing bipolar disorder, the most widely used is the Mood Disorder Questionnaire (MDQ). However, because the MDQ focuses primarily on the retrospective identification of manic or hypomanic episodes, its utility is limited in primary care settings where a depressive episode is often the index manifestation of the illness.

Integrating the robust markers identified in this review—namely early or postpartum onset, frequent recurrences, family history, cyclothymia, mixed or psychotic features, and past suicide attempts—into routine clinical interviews could effectively complement existing screening tools and significantly accelerate the identification of bipolar disorder.

## Conclusion

This review identified the clinical features of depressive episodes that may be indicative of bipolar disorder. The findings show that several factors are frequently associated with bipolar disorder, including early age at onset, recurrent episodes, family history of bipolar disorder, cyclothymic temperament, suicide attempts, postpartum depression, as well as the presence of psychotic features and mixed episodes.

These results suggest that bipolar disorder can be identified at an early stage during a depressive episode. However, the interpretation of these findings should take into account the heterogeneity of the included studies and certain methodological limitations.

Future research should focus on standardising assessment criteria and developing reliable predictive tools to improve early detection of bipolar disorder in primary care settings.

## Supplementary Material

Supplemental Material

## Appendix A. Quality assessment scores using the Newcastle-Ottawa scale

**Table:**

Reference	Selection	Comparability	Exposure	Total
[25]	3	1	2	6/9
[26]	4	1	2	7/9
[27]	3	1	2	6/9
[28]	3	1	2	6/9
[29]	3	1	2	6/9
[30]	4	1	2	7/9
[31]	4	1	2	7/9
[32]	4	2	2	8/9
[33]	4	1	2	7/9
[34]	2	1	2	5/9
[35]	N/A	N/A	N/A	24/27
[36]	2	1	2	5/9
[37]	4	1	2	7/9
[38]	4	2	3	9/9
[39]	3	1	2	6/9
[40]	4	1	2	7/9
[41]	3	1	2	6/9
[42]	4	2	3	9/9
[43]	4	2	2	8/9
[44]	4	1	2	7/9
[45]	3	1	2	6/9
[46]	2	1	1	4/9
[47]	3	1	2	6/9
[48]	4	2	2	8/9
[49]	4	2	3	9/9
[50]	3	1	2	6/9
[51]	4	2	3	9/9
[52]	4	1	2	7/9
[53]	4	1	2	7/9
[54]	2	1	2	5/9
[55]	4	2	2	8/9
[56]	4	1	2	7/9
[57]	4	2	2	8/9
[58]	4	1	2	7/9
[23]	4	2	3	9/9
[59]	4	1	2	7/9
